# Genome-Wide Characterization of the *GmCOPT* Family and Its Salt-Responsive Expression Patterns in Soybean

**DOI:** 10.3390/genes17101293

**Published:** 2026-10-16

**Authors:** Yandang Liu, Lang Pan, Chi Zhang, Shilei Liu, Ning Wang, Kai Zhang, Xinyu Yang, Junyi Gai, Yan Li

**Affiliations:** 1 State Key Laboratory of Crop Genetics & Germplasm Enhancement and Utilization, National Center for Soybean Improvement, Key Laboratory for Biology and Genetic Improvement of Soybean, Jiangsu Collaborative Innovation Center for Modern Crop Production, Nanjing Agricultural University, Nanjing 210095, China; 2 Jiangsu Key Laboratory for the Research and Utilization of Plant Resources, Institute of Botany, Jiangsu Province and Chinese Academy of Sciences (Nanjing Botanical Garden Mem. Sun Yat-Sen), Nanjing 210014, China

**Keywords:** soybean, *COPT* family, copper transporter, salt stress, gene expression

## Abstract

**Background:** Copper transporters (COPTs) are essential for maintaining copper homeostasis in plants, a process critical for growth, development, and stress adaptation. However, the soybean *GmCOPT* family remains poorly characterized. **Methods:** In this study, we performed a genome-wide identification of *COPT* genes in soybean (*Glycine max*), conducted phylogenetic, collinearity, and evolutionary analyses, predicted protein motifs, structures, and promoter *cis*-regulatory elements, and analyzed the expression profiles of the *GmCOPT* family. **Results:** We identified ten *GmCOPT* genes. Phylogenetic analysis of COPT proteins across five plant species revealed that soybean COPTs are more closely related to those from *Medicago truncatula*. Collinearity analysis suggested that segmental duplication is the primary driver of *GmCOPT* family expansion, and all duplicated gene pairs exhibited *Ka/Ks* ratios below 1, indicating purifying selection. Expression profiling revealed tissue-preferential patterns among *GmCOPT* genes. The presence of ABRE, MBS, and LTR *cis*-acting regulatory elements in the promoter regions of *GmCOPTs* suggests their potential involvement in abiotic stress responses. RT-qPCR validation demonstrated that *GmCOPT* genes responded differentially to salt stress in soybean roots and leaves, with some significantly repressed and others markedly induced. Notably, *GmCOPT7* and *GmCOPT8* were significantly induced in both tissues in response to salt stress. **Conclusions:** These findings suggest that *GmCOPT* genes have undergone duplication-driven expansion and functional diversification, with different expression patterns and salt-inducible responses. This work provides candidate genes for future functional investigations into the roles of *GmCOPT* genes in abiotic stress tolerance in soybean.

## 1. Introduction

Soil salinization poses a major constraint on global sustainable agriculture [1]. Under salt stress, excess Na^+^ disrupts cellular ion homeostasis, triggers overproduction of reactive oxygen species (ROS) [2,3], and impairs photosynthesis. These physiological perturbations lead to stunted growth, premature leaf senescence, and substantial yield losses in susceptible crops [4,5,6]. Salinity also inhibits root development and impedes nutrient uptake. Critically, salt stress perturbs the homeostasis of essential micronutrients, including copper (Cu), altering their distribution among plant organs [7]. Cu status affects ABA biosynthesis [8], and ABA signaling restricts Na^+^ accumulation under salt stress [9].

Cu is an essential micronutrient for plant growth and development [10]. As a cofactor of key enzymes such as superoxide dismutase, plastocyanin and cytochrome c oxidase, Cu participates in antioxidant defense, photosynthetic electron transport, and mitochondrial respiration [11,12,13,14]. However, excessive free Cu triggers the Fenton reaction to produce toxic hydroxyl radicals, which inflict irreversible oxidative damage on DNA, proteins, and lipids [15,16,17]. To counter this dual nature, plants have evolved sophisticated Cu trafficking and compartmentalization systems to maintain intracellular Cu homeostasis under both normal and adverse conditions [18].

Copper transporter (COPT) proteins are integral membrane carriers responsible for cellular Cu^+^ uptake and distribution in plants. These transporters facilitate Cu absorption, inter-tissue translocation, and vacuolar Cu sequestration, thereby maintaining Cu homeostasis and mitigating Cu toxicity [19,20,21]. COPT members exhibit diverse and often species-specific functional characteristics across model and crop species. In *Arabidopsis thaliana*, six *AtCOPT* genes regulate high-affinity Cu uptake, vascular transport, and the coordination between Cu and iron homeostasis [19,20,22]. In tomato, *SlCOPT1* and *SlCOPT2* mediate Cu acquisition under Cu deficiency, whereas fungal symbiosis-induced *SlCOPT* expression improves Cu tolerance and drought resistance by restricting root-to-shoot Cu translocation [23,24]. In *M. truncatula*, *MtCOPT* genes support symbiotic nitrogen fixation and mycorrhizal Cu uptake [25,26]. In rice, seven *OsCOPT* members modulate high-affinity Cu absorption, vascular Cu distribution, grain Cu loading, and basal disease resistance, with distinct subcellular-localized isoforms coordinating whole-plant Cu translocation [21,27,28,29]. In maize, three ZmCOPT transporters have been characterized: ZmCOPT1 and ZmCOPT2 exhibit high-affinity Cu^+^ transport activity, and the expression of *ZmCOPT* genes responds to both Cu deficiency and excess [30]. In addition, previous studies have also implicated *COPT* in plant salinity tolerance. Specifically, *COPT* positively regulates salt stress responses by mediating Cu homeostasis and modulating ABA biosynthesis and signaling under copper-sufficient conditions [8]. However, these findings are primarily derived from other species, and whether orthologous genes play similar roles in soybean (*G. max*), an economically important legume crop for edible oil and protein [31], remains unknown. While *COPT* families have been functionally characterized in multiple plant species, the soybean *GmCOPT* family remains poorly understood, and its potential role in soybean response to salt stress has not been systematically investigated.

To address this knowledge gap, we performed a genome-wide identification of the *GmCOPT* family genes and conducted comprehensive analyses encompassing protein properties, interspecific phylogeny, gene duplication events, promoter *cis*-regulatory elements, tissue expression profiles, and expression patterns in response to salt stress. This study systematically characterized the soybean *GmCOPT* family and identified *GmCOPT* candidate genes for further functional studies. Our findings provide a foundation for understanding the potential roles of *COPTs* in soybean response to salinity and offer promising resources for future functional validation.

## 2. Materials and Methods

### 2.1. Identification and Bioinformatic Analysis of the GmCOPT Gene Family in Soybean

Genomic sequences, protein sequences, and GFF3 (Generic Feature Format version 3) annotation files of *G. max* Wm82.a4.v1 were downloaded from Phytozome v14 (https://phytozome-next.jgi.doe.gov/, accessed on 28 June 2026) [32]. The HMM profile of the COPT domain (PF04145) was obtained from Pfam database (https://www.ebi.ac.uk/interpro/entry/pfam/#table/, accessed on 28 June 2026) [33]. HMM searches were performed against the whole proteomes of *G. max*, *A. thaliana*, *M. truncatula*, *Oryza sativa* and *Zea mays* using TBtools-II [34] with an E-value cutoff of 1 × 10^−5^ to identify COPT proteins [35]. After redundant sequences were eliminated, all candidate proteins were verified using NCBI CDD database (https://www.ncbi.nlm.nih.gov/Structure/cdd/cdd.shtml, accessed on 28 June 2026) [36] to confirm the presence of the intact PF04145 domain. Only proteins containing this intact domain were retained as final COPT candidates, yielding ten GmCOPT proteins from soybean, six from *A. thaliana,* eight from *M. truncatula*, six from *O. sativa*, and seven from *Z. mays*. The physicochemical properties, including amino acid length, molecular weight, and theoretical pI, were calculated using ExPASy ProtParam (https://web.expasy.org/protparam, accessed on 28 June 2026) [37] (Table S1), and their subcellular localizations were predicted using WoLF PSORT (https://wolfpsort.hgc.jp/, accessed on 28 June 2026) [38].

### 2.2. Phylogenetic Tree Construction

Multiple sequence alignment was performed using MAFFT online server (https://mafft.cbrc.jp/alignment/server/, accessed on 16 September 2026). A circular phylogenetic tree was constructed using the Maximum Likelihood (ML) method implemented in MEGA 7.0 [39], with the JTT+G amino acid substitution model identified as optimal based on the Bayesian Information Criterion (BIC). Bootstrap analysis was performed with 1000 replicates.

### 2.3. Collinearity and Evolutionary Analysis

Genomic sequences and GFF3 gene annotation files of *G. max* and *A. thaliana* were downloaded from the EnsemblPlants database (https://plants.ensembl.org/index.html, accessed on 3 July 2026) [40]. The *G. max* assembly *Glycine_max*_v2.1 and the *A. thaliana* TAIR10 reference genome were used in this study. The correspondence of the 10 *GmCOPT* gene models between the Wm82.a4.v1 (used for gene identification) and *Glycine_max*_v2.1 assemblies, together with their chromosomal coordinates, is provided in Table S2. Intraspecies collinearity analysis of the *GmCOPT* gene family and interspecies whole-genome synteny analysis between soybean and *Arabidopsis* were conducted using the One-step MCScanX toolkit integrated in TBtools-II [34,41]. Collinear homologous gene pairs between *GmCOPTs* and the *A. thaliana COPT* genes were identified, and coordinate information of the corresponding collinear blocks was extracted. Circular and linear collinearity plots were generated using the advanced visualization modules of TBtools-II. In addition, the non-synonymous (*Ka*) and synonymous (*Ks*) substitution rates of duplicated gene pairs were calculated using the simple *Ka/Ks* calculator embedded in TBtools-II.

### 2.4. Analysis of Protein Motifs and Structures

Conserved motifs of GmCOPT protein sequences were identified using the MEME web server (https://meme-suite.org/meme/tools/meme, accessed 29 June 2026) [42], and the distribution patterns of these motifs were visualized using TBtools-II [40]. The three-dimensional structures of GmCOPT proteins were predicted using the SWISS-MODEL web platform (https://swissmodel.expasy.org/interactive, accessed 2 August 2026). The quality of the predicted models was assessed using GMQE [43], MolProbity [44], Ramachandran plot analysis, and clash scores [45], and the detailed metrics are provided in Table S3.

### 2.5. Analysis of Cis-Acting Elements in Promoter Regions of GmCOPT Genes

To identify putative *cis-*acting elements, the 2000 bp genomic sequences immediately upstream of the start codon of each *GmCOPT* gene were extracted from the Wm82.a4.v1 assembly using TBtools-II and designated as putative promoter regions. These sequences were then submitted to the PlantCARE online database (http://bioinformatics.psb.ugent.be/webtools/plantcare/html/, accessed on 30 June 2026) [46] for prediction of *cis-*elements. The distribution patterns and abundance of these *cis*-elements across the *GmCOPT* promoters were visualized as a heatmap using TBtools-II.

### 2.6. Tissue Expression Profiling

FPKM (Fragments Per Kilobase of transcript per Million mapped reads) values for different soybean tissues were retrieved from the Phytozome database (https://phytozome-next.jgi.doe.gov/, accessed on 29 June 2026), specifically from the DOE Joint Genome Institute (JGI) Plant Gene Atlas project [47], based on the *G. max* cv. Williams 82 reference genome. Raw RNA-seq data are available at NCBI under BioProject PRJNA372533. Seven tissues were included: roots, nodules, stems, leaves, flowers, pods, and seeds, with three biological replicates per sample group. Raw reads were processed using the standardized JGI pipeline (aligned with GSNAP and quantified with HTSeq v1.99.2), and FPKM values were calculated by normalizing read counts to gene length and total mapped reads. Since the raw count matrices per replicate were not available in the Phytozome module, no additional statistical tests were performed on these aggregated FPKM values. These normalized values were used to assess the tissue expression patterns of the target genes.

### 2.7. Plant Materials, Salt Stress Treatment, and Sample Collection

Soybean cultivar Tianlong 1 was used in this study. Soybean seeds were provided by the National Center for Soybean Improvement, Nanjing Agricultural University. Seeds were surface-sterilized with 75% ethanol for 30 s and rinsed three times with sterile distilled water. They were then germinated in sand for three days. Seedlings with uniform growth were transplanted into beakers containing half-strength Hoagland’s solution (Hopebiol Biotechnology, Qingdao, China) and cultivated in a greenhouse at 26 °C with 75% relative humidity under a 16 h light/8 h dark photoperiod. When the first trifoliate leaf was fully expanded, seedlings were treated with half-strength Hoagland’s solution (pH 5.8) supplemented with either 0 mM NaCl (control) or 200 mM NaCl, following a previously reported salt treatment protocol with minor modifications [48], in which soybean seedlings were treated with 200 mM NaCl. In the present study, root and leaf tissues were harvested at 6, 12, and 24 h post-treatment, with three biological replicates, each consisting of a pooled sample of two individual plants. For each time point, both 0 mM and 200 mM NaCl samples were collected, and the 0 mM NaCl samples served as time-matched controls for the corresponding 200 mM NaCl samples.

### 2.8. RNA Extraction and Reverse-Transcription Quantitative PCR (RT-qPCR) Analysis

Total RNA was extracted using the RNApure Fast Plant Kit (CW0598S, CWBlO, Taizhou, Jiangsu, China) according to the manufacturer’s instructions. RNA quality was assessed using a NanoDrop One spectrophotometer (Thermo Fisher Scientific, Waltham, MA, USA), and the A260/280 and A260/230 ratios for all samples are provided in Table S4. Complementary DNA (cDNA) was synthesized using HiFiScript All-in-one RT Master Mix for qPCR Kit (CW3371, CWBlO, Taizhou, Jiangsu, China). RT-qPCR was performed using SuperStar Blue Universal SYBR Master Mix (CW3390, CWBlO, Taizhou, Jiangsu, China) on a LightCycler480 System (Roche Diagnostics, Rotkreuz, Switzerland). Each 15 μL reaction mixture contained 5.7 μL of ddH_2_O, 1.2 μL of cDNA, 0.3 μL each of 10 μM forward and reverse primers, and 7.5 μL of 2 × SuperStar Blue Universal SYBR Master Mix. The amplification protocol included initial denaturation at 95 °C for 3 min, followed by 40 cycles of annealing and extension at 60 °C for 30 s. Melting curve analysis confirmed a single melting peak for all primer pairs (Figure S1). Primer sequences and efficiencies are provided in Table S5. Primer efficiency was determined by standard curve analysis using a 2-fold dilution series of cDNA [49] and calculated as: efficiency = (10^−1/slope^ − 1) × 100 [50]. *Gm60S* was used as the internal reference gene [51], with stability validated across all samples (CV% < 5%), confirming its suitability for normalization (Table S6) [52]. Relative gene expression was calculated using the 2^−ΔΔCt^ method [53], with the time-matched 0 mM NaCl sample used as the calibrator for each time point. Raw RT-qPCR data, including Ct and ΔCt values, are provided in Table S7.

### 2.9. Statistical Analysis

All experiments were performed with three independent biological replicates. Statistical comparisons between the control (0 mM NaCl) and salt treatment (200 mM NaCl) groups were performed using two-tailed Student’s *t*-test in Microsoft Excel. Differences were considered significant at *p* < 0.05 (*) and *p* < 0.01 (**). Graphs were generated using GraphPad Prism 8.0.2 (GraphPad Software, Boston, MA, USA).

## 3. Results

### 3.1. Genome-Wide Identification and Phylogenetic Analysis of the GmCOPT Gene Family

Using the conserved COPT domain (PF04145), a genome-wide Hidden Markov model (HMM) screening identified ten soybean *COPT* genes, which were designated *GmCOPT1* to *GmCOPT10* based on their chromosomal locations (Table S1). The encoded GmCOPT proteins ranged from 135 to 155 amino acids in length, with molecular weights spanning 14,849.64 to 16,927.20 Da, and all were predicted to be alkaline proteins. Subcellular localization predictions revealed diverse compartmental distributions among the family members: GmCOPT1, GmCOPT4, GmCOPT5, GmCOPT6, GmCOPT9, and GmCOPT10 were predicted to localize to the cell membrane; GmCOPT2 and GmCOPT7 were predicted to localize to both chloroplast and Golgi apparatus; GmCOPT3 was predicted to localize to the chloroplast; and GmCOPT8 was predicted to reside in the cell membrane, chloroplast, and Golgi apparatus (Table S1). These predictions require further experimental validation.

**Figure 1:**
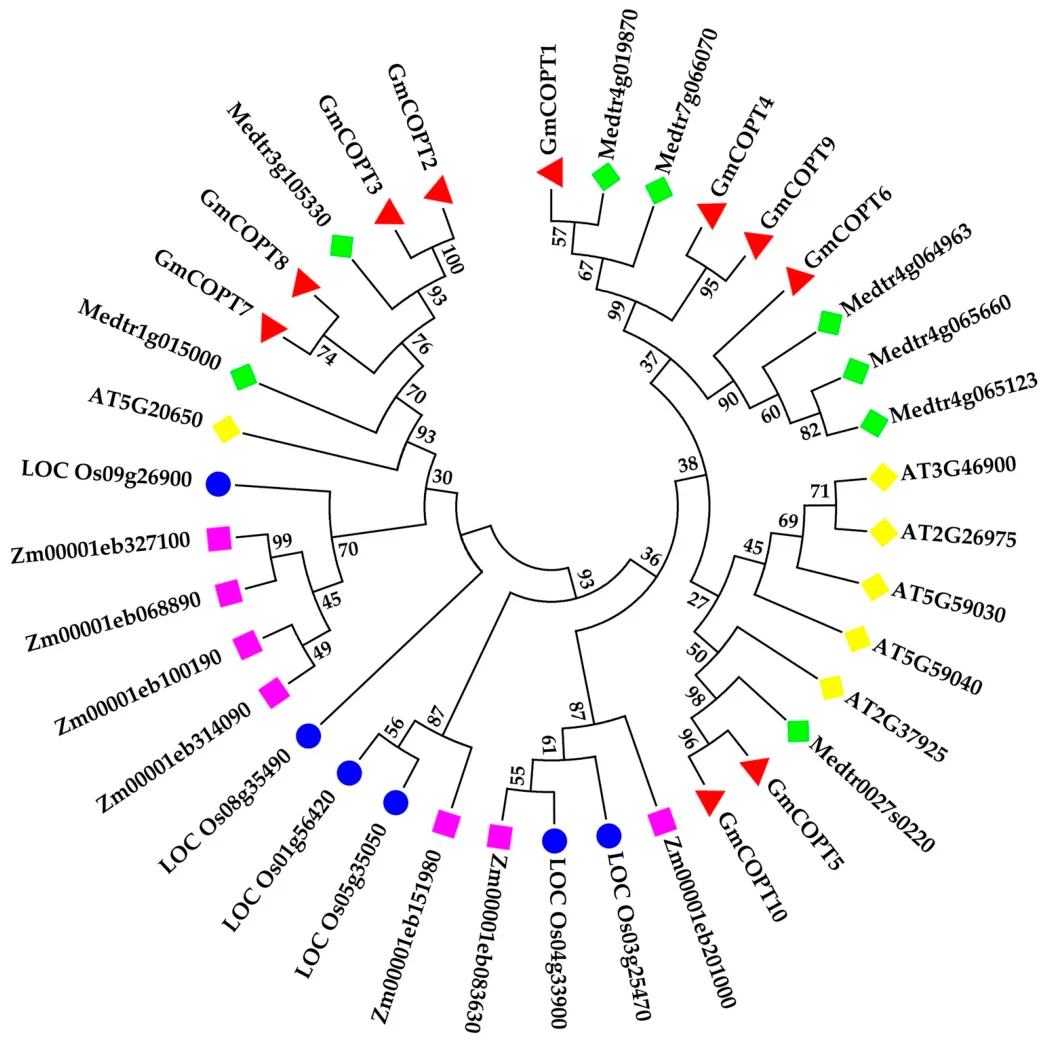
Phylogenetic tree of COPT proteins from five plant species. Red triangles, *G. max*; green diamonds, *M. truncatula*; yellow diamonds, *A. thaliana*; blue circles, *O. sativa*; magenta squares, *Z. mays*. Bootstrap values are shown at each branch node.

To investigate the evolutionary relationships of COPT proteins, we constructed a phylogenetic tree (Figure 1) based on multiple sequence alignments of COPTs from five species (Table S8), including *G. max* (Gm), *A. thaliana* (AT), *M. truncatula* (Medtr), *O. sativa* (LOC Os), and *Z. mays* (Zm). The soybean GmCOPT proteins (GmCOPT1–GmCOPT10) are distributed across multiple clades rather than clustering into a single soybean-specific clade, indicating the GmCOPT family may have undergone functional diversification. GmCOPTs are most closely related to *M. truncatula* COPT proteins, consistent with their shared legume ancestry. Five of the six *Arabidopsis* COPT proteins (AT3G46900, AT2G26975, AT5G59030, AT5G59040, AT2G37925) are more closely related to Medtr0027s0220 and GmCOPT5/10, whereas AT5G20650 is closer to Medtr1g015000, GmCOPT2/3, Medtr3g105330 and GmCOPT7/8. COPTs from rice and maize are more closely related to each other. These results indicate that *GmCOPT* genes maintain a close evolutionary relationship with *M. truncatula* COPTs.

### 3.2. Collinearity and Evolutionary Analysis of the GmCOPT Gene Family

**Figure 2:**
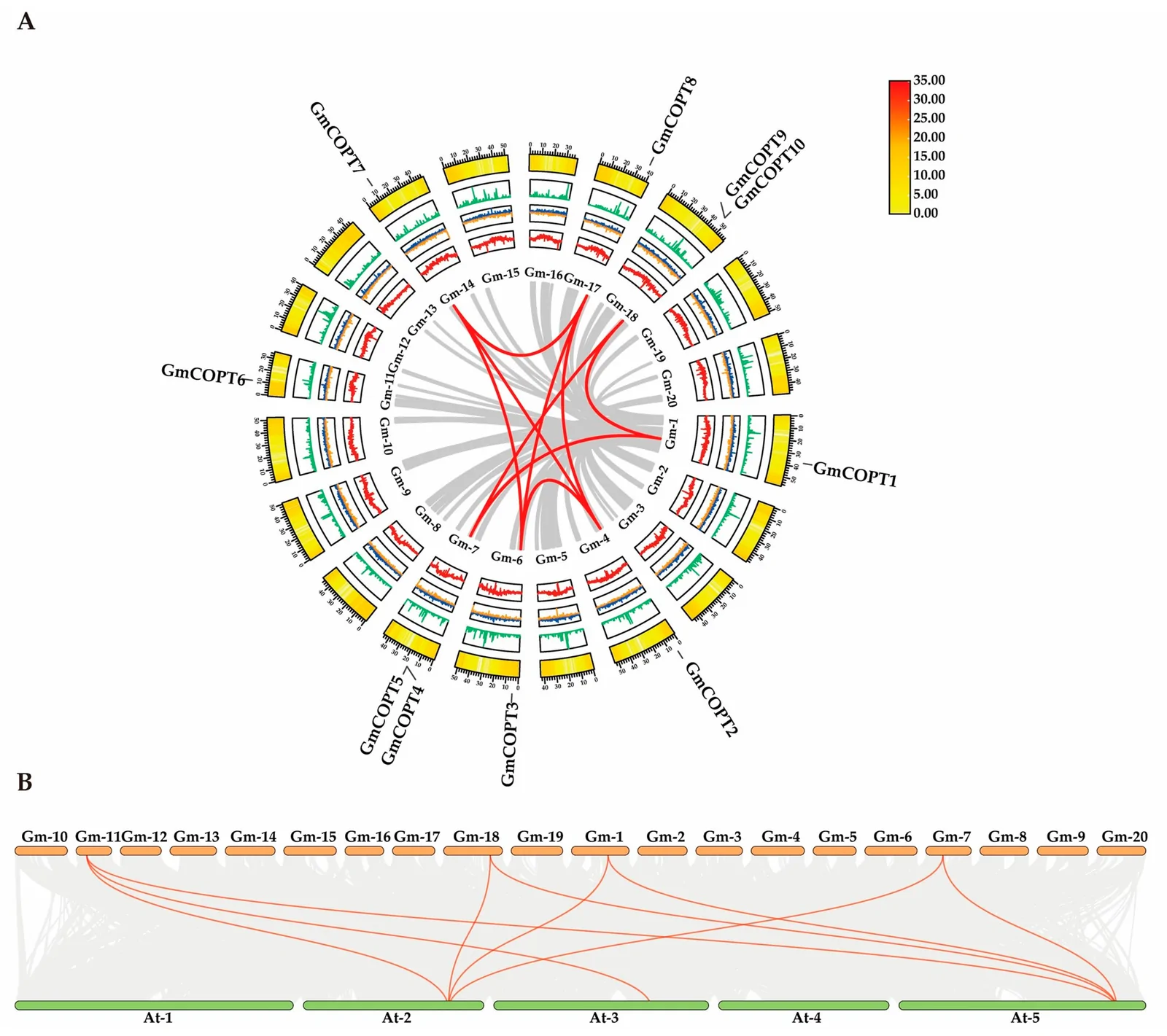
Collinearity analysis of the *COPT* gene family within soybean and between soybean and *Arabidopsis. (***A**) Intraspecies collinearity of *GmCOPT* genes across soybean chromosomes. From the outermost to the innermost rings, the circular plot sequentially displays gene density (yellow-to-red heatmap), N ratio (green lines), GC skew (blue and orange lines), GC ratio (red lines), soybean chromosomes, and genome-wide collinear blocks. Gray lines represent all collinear gene blocks across the soybean genome, whereas red lines highlight paralogous *GmCOPT* gene pairs located within conserved collinear blocks. (**B**) Interspecies collinearity of *COPT* genes between *G. max* and *A. thaliana*. Orange horizontal bars represent soybean chromosomes (Gm1–Gm20), while green horizontal bars denote the five *A. thaliana* chromosomes (At1–At5). Orange lines connect orthologous *COPT* gene pairs that exhibit conserved collinearity between the two species.

To investigate the evolutionary mechanisms underlying the expansion of the *GmCOPT* family, we performed genome-wide collinearity analysis (Figure 2). A total of nine pairs of segmentally duplicated genes were identified (Figure 2A, Table S9), whereas no tandem duplication events were detected, suggesting that segmental duplication is the primary driver of *GmCOPT* family expansion. All paralogous pairs exhibited *Ka/Ks* < 1 (Table S9), indicating they have evolved under purifying selection, with the *GmCOPT2/GmCOPT3* pair displaying the strongest evolutionary constraint (lowest *Ka/Ks*, Table S9). Cross-species synteny between soybean and *Arabidopsis* revealed that five soybean genes—*GmCOPT1*, *GmCOPT4*, *GmCOPT6*, *GmCOPT9*, and *GmCOPT10*—are orthologous to *AT2G37925*, *AT3G46900*, and *AT5G59030* (Figure 2B). This suggests that the ancestral *COPT* gene originated prior to the divergence of dicotyledonous plants and that subsequent ancient polyploidization events contributed to expanded gene copies. Among the orthologous pairs with calculable *Ka/Ks* values, the ratios ranged from 0.1168 to 0.2361 (Table S10), consistent with purifying selection. Other orthologous pairs failed to yield valid *Ks* estimates, likely due to synonymous substitution saturation. Collectively, these results demonstrate that segmental duplication dominates the expansion of the *GmCOPT* family. Furthermore, both intra-species paralogs and inter-species orthologs are subject to purifying selection.

### 3.3. Conserved Motifs and Predicted Three-Dimensional Structures of GmCOPT Proteins

**Figure 3:**
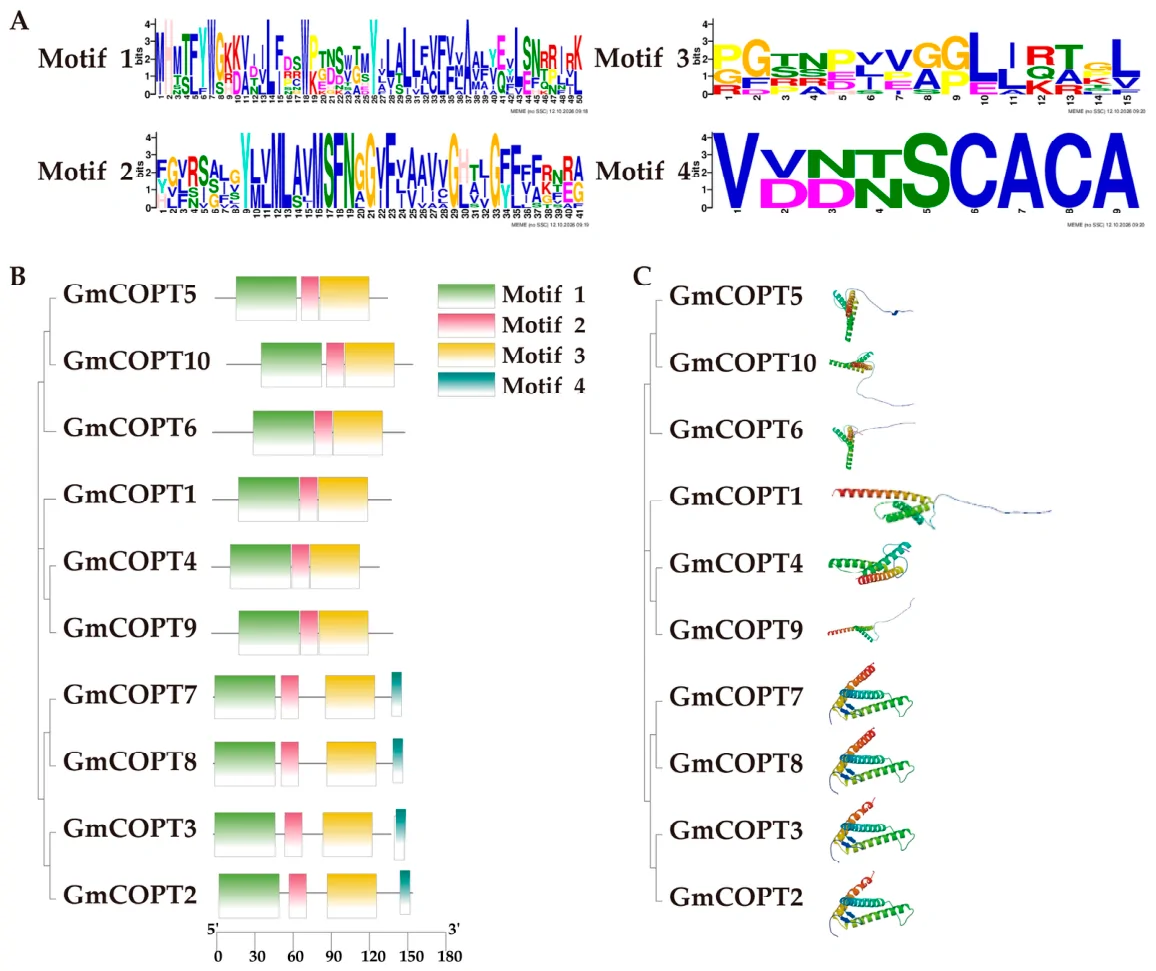
Conserved motifs and predicted three-dimensional structures of GmCOPT proteins. (**A**) Sequence logos of four conserved motifs (Motif 1–4) identified by MEME. (**B**) Distribution of the conserved motifs in each GmCOPT protein. (**C**) Predicted three-dimensional structures of GmCOPT proteins generated using SWISS-MODEL (https://swissmodel.expasy.org).

MEME analysis identified four conserved motifs (Motifs 1–4) among the ten GmCOPT proteins (Figure 3A, Table S11). Motifs 1–3 were present in all GmCOPT members, whereas Motif 4 was exclusively detected in the phylogenetically closely related members GmCOPT2, GmCOPT3, GmCOPT7, and GmCOPT8 (Figure 3B). Predicted three-dimensional (3D) structures revealed that closely related GmCOPT paralogs display more similar 3D structures, whereas distinct 3D structures were observed between phylogenetically divergent groups (Figure 3C). Collectively, the differential motifs and 3D protein structures suggest functional diversification of GmCOPT proteins, which requires future experimental validation.

### 3.4. Cis-Acting Elements Analysis in the Promoter Regions of GmCOPT Genes

**Figure 4:**
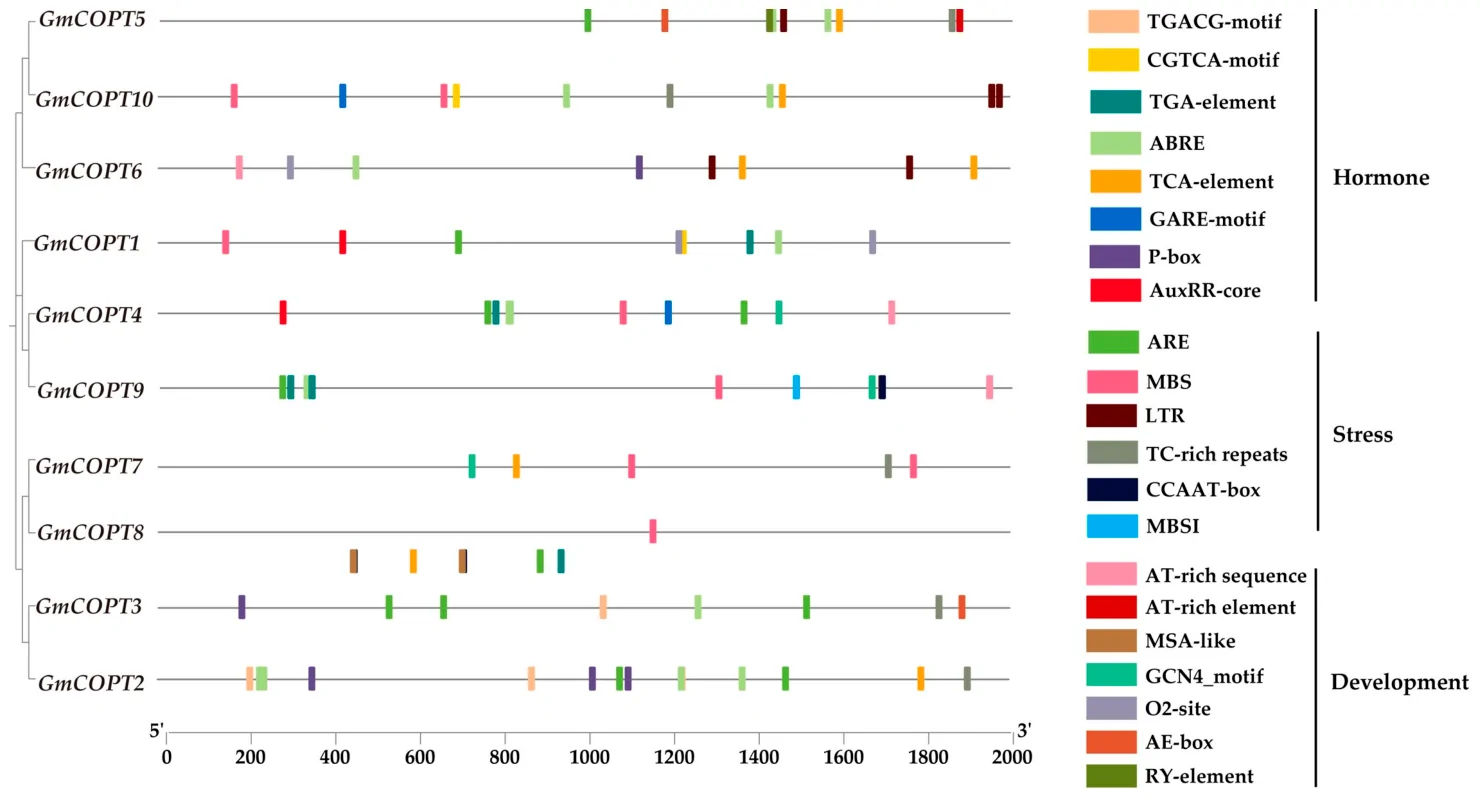
Distribution of predicted *cis*-acting regulatory elements in the 2000 bp promoter regions upstream of the start codons of *GmCOPT1*–*GmCOPT10*. Colored vertical bars represent different *cis*-elements, with horizontal positions denoting their locations in each promoter. The legend on the right provides the names of the predicted elements. Light-responsive elements are listed in Table S13.

**Figure 5:**
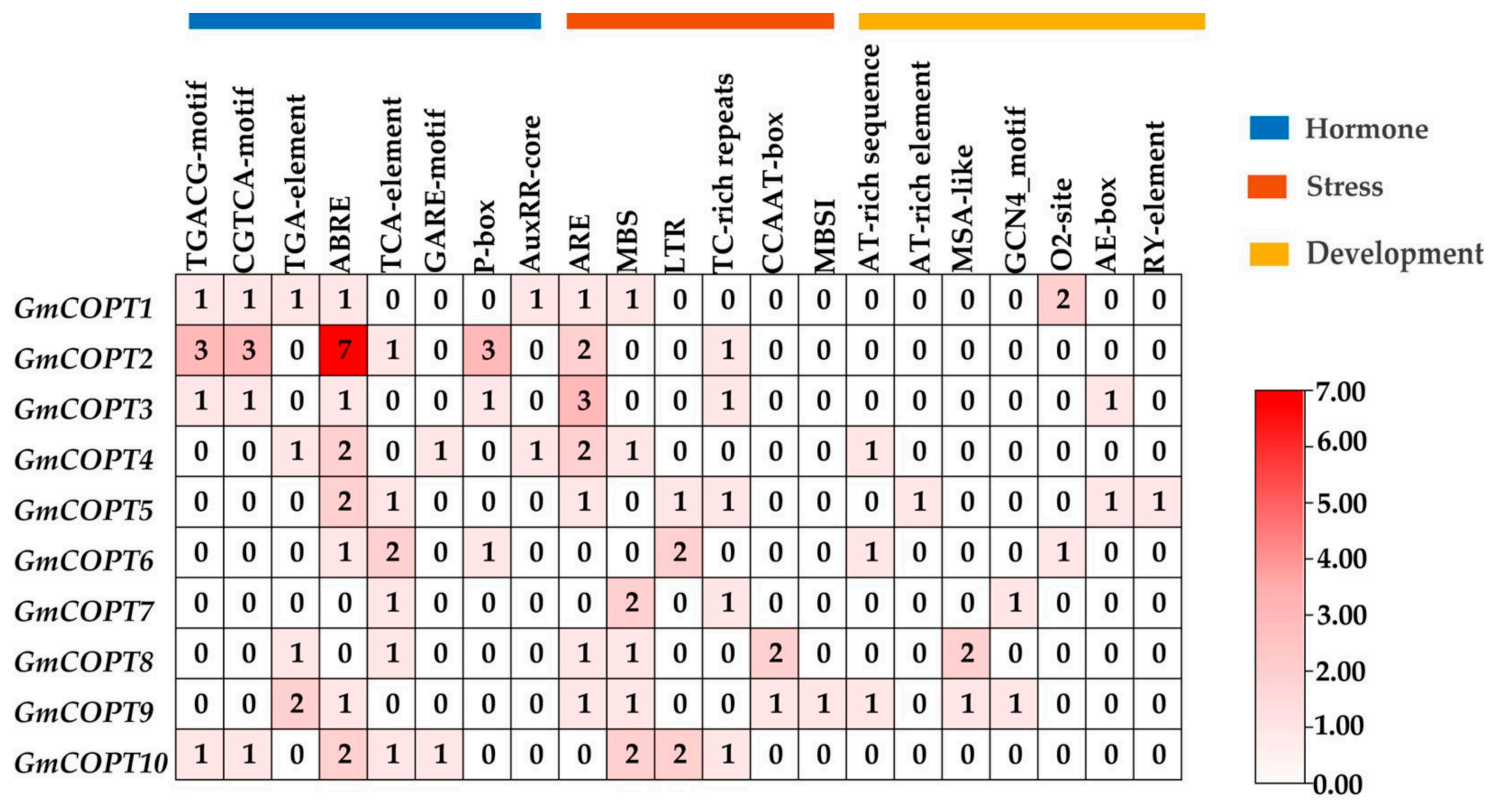
Heatmap displaying the abundance of various *cis*-acting regulatory elements predicted in the promoter regions of ten soybean *GmCOPT* genes. Each column represents a specific *cis*-element, and each row corresponds to an individual *GmCOPT* gene. Numerical values indicate the copy numbers of each *cis*-element present in the respective promoter region. The color gradient from white to deep red corresponds to an increase in copy number. The *cis*-elements are classified into three functional categories in this figure (hormone-responsive, stress-responsive, and development-associated elements), as indicated by the colored bars at the top of the heatmap. Light-responsive elements are listed in Table S13.

Given the critical roles of *cis*-acting elements in gene regulation, we analyzed their distributions within the 2000 bp promoter regions upstream of the start codons of ten *GmCOPT* genes (Figure 4 and Figure 5, Table S12 and Table S13). The predicted elements were classified into three major categories: hormone-responsive, stress-responsive, and development/light-responsive elements. Hormone-responsive elements, including ABRE, TGACG-motif, TCA-element, GARE-motif, and others, were broadly detected across the promoters. Predicted stress-related motifs were also commonly observed, such as the drought-responsive MBS, the anaerobic-responsive element ARE, and the cold-inducible LTR. In addition, light-responsive *cis*-acting elements, such as G-box, were identified in the promoter regions of *GmCOPTs* (Table S13). The development-related element O2-site was identified in the promoters of several *GmCOPTs*. Notably, these predicted *cis*-elements appeared to be randomly scattered throughout the promoter regions, without fixed conserved positions (Figure 4).

Further analysis revealed that the composition and abundance of predicted *cis*-elements varied among *GmCOPT*s (Figure 5). Each *GmCOPT* gene exhibited a unique *cis*-element signature, suggesting that they may be differentially regulated in response to specific environmental cues or developmental signals. The presence of predicted ABRE (ABA-responsive), MBS (drought-related MYB binding site), and LTR (cold-inducible) elements in *GmCOPT* promoters suggests that these genes may be responsive to abiotic stress. However, this remains predictive and requires functional validation, such as promoter-reporter assays or transcription factor binding experiments.

### 3.5. Tissue Expression Pattern of GmCOPT Genes

To investigate the spatial expression profiles of the ten *GmCOPT* genes, their transcript abundances were examined across seven soybean tissues, including roots, nodules, stems, leaves, flowers, pods, and seeds (Table S14). Different tissue-preferential expression patterns were observed among the family members. Specifically, *GmCOPT1* exhibited high transcript levels in flowers, followed by nodules and leaves; *GmCOPT2* was predominantly expressed in flowers, followed by stems, seeds, and roots; *GmCOPT6* showed the strongest expression in leaves. In contrast, *GmCOPT4* maintained nearly undetectable expression across most tissues. The remaining genes—*GmCOPT3*, *GmCOPT5*, and *GmCOPT7* through *GmCOPT10*—displayed low-to-moderate expression levels in the examined tissues. These divergent tissue expression patterns suggest that different *GmCOPT* members may be functionally specialized in distinct soybean organs, which requires further experimental validation.

### 3.6. Expression Responses of GmCOPT Genes to Salt Stress

**Figure 6:**
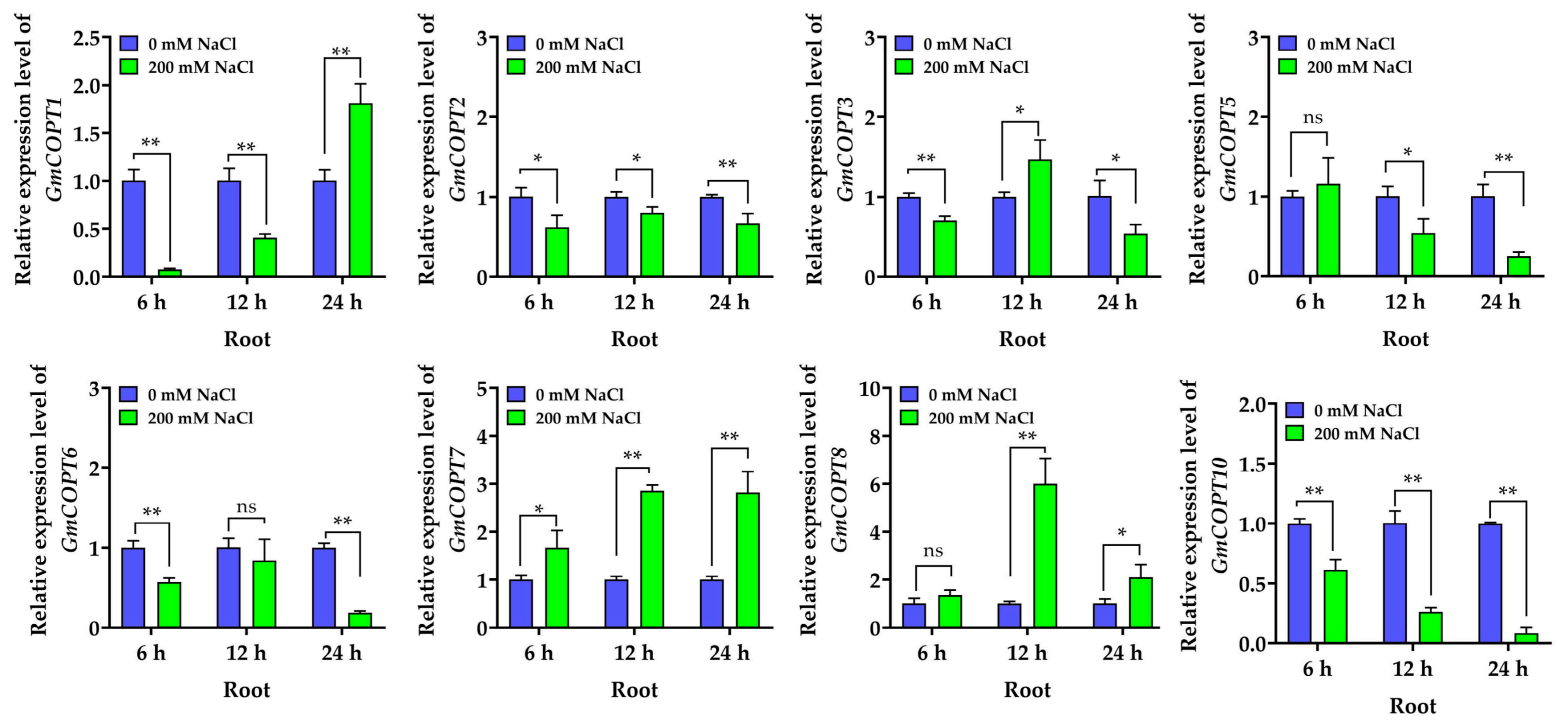
Relative expression levels of *GmCOPT* genes in soybean roots in response to salt stress. RT-qPCR was used to quantify the transcript abundance of *GmCOPT* genes in soybean roots after 6, 12, and 24 h of salt treatment. The 0 mM NaCl sample at each time point was used as the control. *Gm60S* was used as the reference gene. Data are presented as mean ± standard deviation (*n* = 3 biological replicates). Significant differences between the salt treatment and the corresponding 0 mM control at each time point were determined using two-tailed Student’s *t*-tests; ** *p* < 0.01; * *p* < 0.05; ns, not significant.

**Figure 7:**
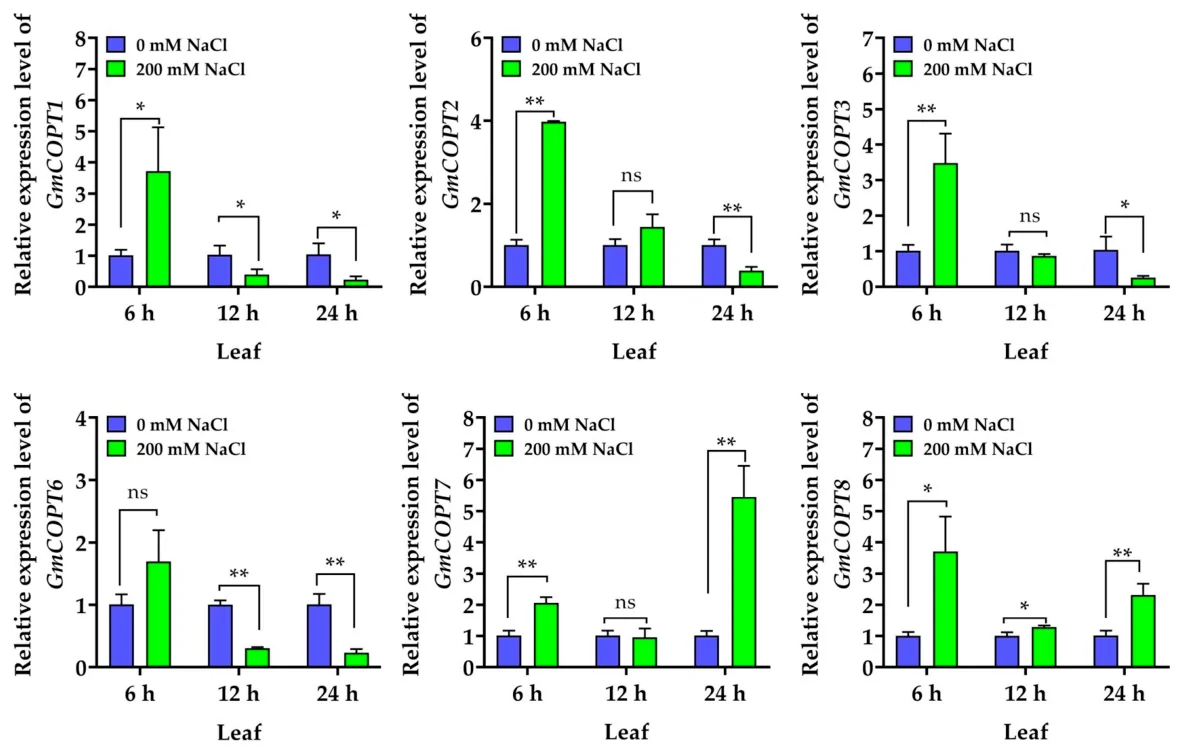
Relative expression levels of *GmCOPT* genes in soybean leaves in response to salt stress. RT-qPCR was used to quantify the transcript abundance of *GmCOPT* genes in soybean leaves after 6, 12, and 24 h of salt treatment. The 0 mM NaCl sample at each time point was used as the control. *Gm60S* was used as the reference gene. Data are presented as mean ± standard deviation (*n* = 3 biological replicates). Significant differences between the salt treatment and the corresponding 0 mM control at each time point were determined using two-tailed Student’s *t*-tests; ** *p* < 0.01; * *p* < 0.05; ns, not significant.

To examine the transcriptional responses of *GmCOPT* genes to salt stress, we quantified their transcript abundances in roots and leaves at 6, 12, and 24 h after salt treatment, along with their corresponding control (0 mM NaCl) at the same time points, using RT-qPCR (Figure 6 and Figure 7). *GmCOPT4* and *GmCOPT9* exhibited low or undetectable transcripts in roots, while *GmCOPT4*, *GmCOPT5*, *GmCOPT9* and *GmCOPT10* showed low or undetectable expression in leaves, which is consistent with the tissue expression profile (Table S14). In roots (Figure 6), *GmCOPT2* and *GmCOPT10* were significantly downregulated at 6, 12 and 24 h in response to salt stress, whereas *GmCOPT7* was induced at all three time points. In leaves (Figure 7), *GmCOPT7* was significantly induced at 6 h and 24 h, while *GmCOPT8* was induced across all three time points in response to salt treatment. These results demonstrate that *GmCOPT* family members respond differentially to salt treatment, and their potential roles in soybean adaptation to salt stress require further functional studies. 

## 4. Discussion

Soil salinization is a major abiotic stress factor that constrains soybean yield and quality improvement. Cu participates in plant photosynthesis and antioxidant metabolism, and Cu homeostasis is essential for plant growth and stress adaptation [18]. Salt stress-induced imbalances in micronutrient homeostasis aggravate oxidative damage and inhibit plant growth [54]. Although *COPT* genes have been characterized in model plants and major crops, including *Arabidopsis*, rice, and tomato, the soybean *GmCOPT* family remains poorly understood. In this study, we systematically characterized the phylogenetic relationships, gene expansion patterns, evolutionary selection pressures, tissue expression profiles, and spatiotemporal transcriptional responses to salt stress of the soybean *GmCOPT* family, thereby filling a critical knowledge gap in soybean.

Segmental duplication and evolutionary selection are core drivers shaping the expansion, structural conservation, and functional divergence of plant gene families [55]. Consistent with previous analyses of the *COPT* family across 53 Chlorophyta and terrestrial plant species [56], the expansion of the soybean *GmCOPT* family is primarily driven by segmental duplication, with all paralogous and orthologous gene pairs subject to purifying selection. In contrast to the conserved single subcellular localization of COPT proteins in *Arabidopsis* and gramineous crops [57], soybean GmCOPT members were predicted to exhibit diversified subcellular localizations, targeting the cell membrane, chloroplasts, and Golgi apparatus. This evolutionary divergence suggests that GmCOPTs may have differentiated functions that require future investigation in soybean.

Previous studies have shown that AtCOPT proteins in *Arabidopsis* primarily mediate high-affinity Cu uptake and vascular Cu translocation under Cu-deficient conditions [58], whereas rice *COPT* genes are specifically induced in response to heavy metal stress. Our study showed that the promoter regions of soybean *GmCOPT* members contain abundant predicted *cis*-elements responsive to phytohormones and stresses, implying that soybean *GmCOPT* genes might be associated with phytohormone signaling and salt stress responses. Furthermore, maize *COPT* genes exhibit constitutive expression across various plant tissues [30], whereas soybean *GmCOPT* genes display pronounced tissue-preferential expression patterns. Specifically, *GmCOPT1* was predominantly expressed in flowers, followed by nodules and leaves, whereas *GmCOPT2* was predominantly expressed in flowers, followed by stems, seeds, and roots; *GmCOPT6* showed the highest expression in leaves. These distinctive spatial expression profiles demonstrate that *GmCOPT* paralogs may have undergone expression divergence during evolution.

*GmCOPTs* responded to salt stress differently between roots and leaves. In roots, *GmCOPT7* and *GmCOPT8* were significantly induced, particularly at 12 and 24 h, while most other detectable *GmCOPT* genes were suppressed. In leaves, multiple *GmCOPTs* showed induction at 6 h after salt stress, and *GmCOPT7* was significantly upregulated at 6 and 24 h. These results suggest *GmCOPT* genes respond to salt stress in a spatiotemporal manner, coinciding with the distinct functions of *COPTs* in different organs to mediate copper redistribution—*AtCOPT1*(*AT5G59030*) in root Cu acquisition, *AtCOPT6* (*AT2G26975*) in Cu distribution to shoots, and *AtCOPT5* (*AT5G20650*) in mobilizing organelle-stored Cu [59,60]. These processes are part of the network that maintains Cu homeostasis [56]. Since copper is an essential cofactor of Cu/Zn-SOD [61], maintaining Cu homeostasis is critical for antioxidant defense under oxidative stress [62], which might contribute to salt response regulation. However, these hypotheses require further experimental validation. *GmCOPTs* were not located in the known salt-tolerance-related QTLs (Table S15) in SoyBase (https://www.soybase.org/), suggesting that they are not the causal genes of these QTLs.

In this study, we verified the transcriptional responses of *GmCOPT* genes to salt stress at the whole-gene-family level. Nevertheless, the specific functions and molecular regulatory mechanisms of each member remain to be further elucidated. Future investigations could focus on functional validation of key candidate genes, including characterization of Cu transport activity of GmCOPT proteins, Cu measurements, phenotypic analyses of overexpression or knockout lines under salt stress, and exploration of upstream regulators and downstream interacting partners. Further studies on these genes will deepen our systematic understanding of abiotic stress tolerance mechanisms in soybean and provide more precise genetic targets for breeding salt-tolerant soybean varieties.

## 5. Conclusions

In summary, we systematically characterized the *GmCOPT* gene family in soybean, revealing segmental duplication-driven expansion and purifying selection. Promoter and expression analyses suggested complex transcriptional regulation, tissue-preferential expression patterns, and salt-inducible responses, with *GmCOPT7* and *GmCOPT8* identified as key salt stress-responsive candidates, implying potential functional divergence among *GmCOPT* paralogs. This study provides a foundation for future functional studies on *GmCOPT* genes in Cu homeostasis and stress tolerance, with potential implications for breeding salt-tolerant soybean varieties.

## Data Availability

The data presented in this study are available in the article/Supplementary Materials. Further inquiries can be directed to the corresponding author.

## Supplementary Materials

The following supporting information can be downloaded at: https://www.mdpi.com/article/10.3390/genes17101293/s1, Table S1. Physicochemical properties of soybean GmCOPT proteins. Table S2. Gene IDs and chromosomal locations of soybean *GmCOPT* genes in the Wm82.a4.v1 and *Glycine_max*_v2.1 genome assemblies. Table S3. Quality assessment of the SWISS-MODEL predicted structures for GmCOPT proteins. Table S4. RNA quality assessment for RT-qPCR samples. Table S5. Primer sequences and efficiencies for RT-qPCR in this study. Table S6. Stability validation of the reference gene *Gm60S* in soybean leaf and root tissues. Table S7. RT-qPCR raw data (Ct and ΔCt values) for *GmCOPT* genes in soybean leaves and roots. Table S8. List of *COPT* genes in *Arabidopsis thaliana*, *Medicago truncatula*, *Oryza sativa* and *Zea mays*. Table S9. *Ka/Ks* values of paralogous *GmCOPT* gene pairs in soybean. Table S10. *Ka/Ks* values of orthologous pairs between soybean *GmCOPT* and the *Arabidopsis COPT* genes. Table S11. Conserved protein motifs of GmCOPTs predicted by MEME. Table S12. Positions of *cis*-acting regulatory elements in the promoter regions of *GmCOPT* genes. Table S13. Positions of light-responsive *cis*-acting regulatory elements in the promoter regions of *GmCOPT* genes. Table S14. Tissue expression profiles of *GmCOPT* genes across seven soybean tissues. Table S15. Known QTLs associated with soybean salt tolerance in SoyBase. Figure S1. Melting curve analysis of the primer pairs used for RT-qPCR. References [63,64,65,66,67,68] are cited in the Supplementary Materials.

## Abbreviations

The following abbreviations are used in this manuscript:

ABA	Abscisic acid
COPT	Copper (Cu) transporter
FPKM	Fragments per kilobase per million mapped reads
HMM	Hidden Markov model
MBS	MYB binding site
RT-qPCR	Reverse transcription quantitative PCR
