# Supplementary material for: Genome-Wide Characterization of the GmCOPT Family and Its Salt-Responsive Expression Patterns in Soybean
Source: Genes (Basel). 2026 Oct 16;17(10):1293. doi: 10.3390/genes17101293 (PMC13704279; doi:10.3390/genes17101293)
Supplement: Supplementary file 1 [file genes-17-01293-s001.zip › Supplementary Figure 20260929.pdf]

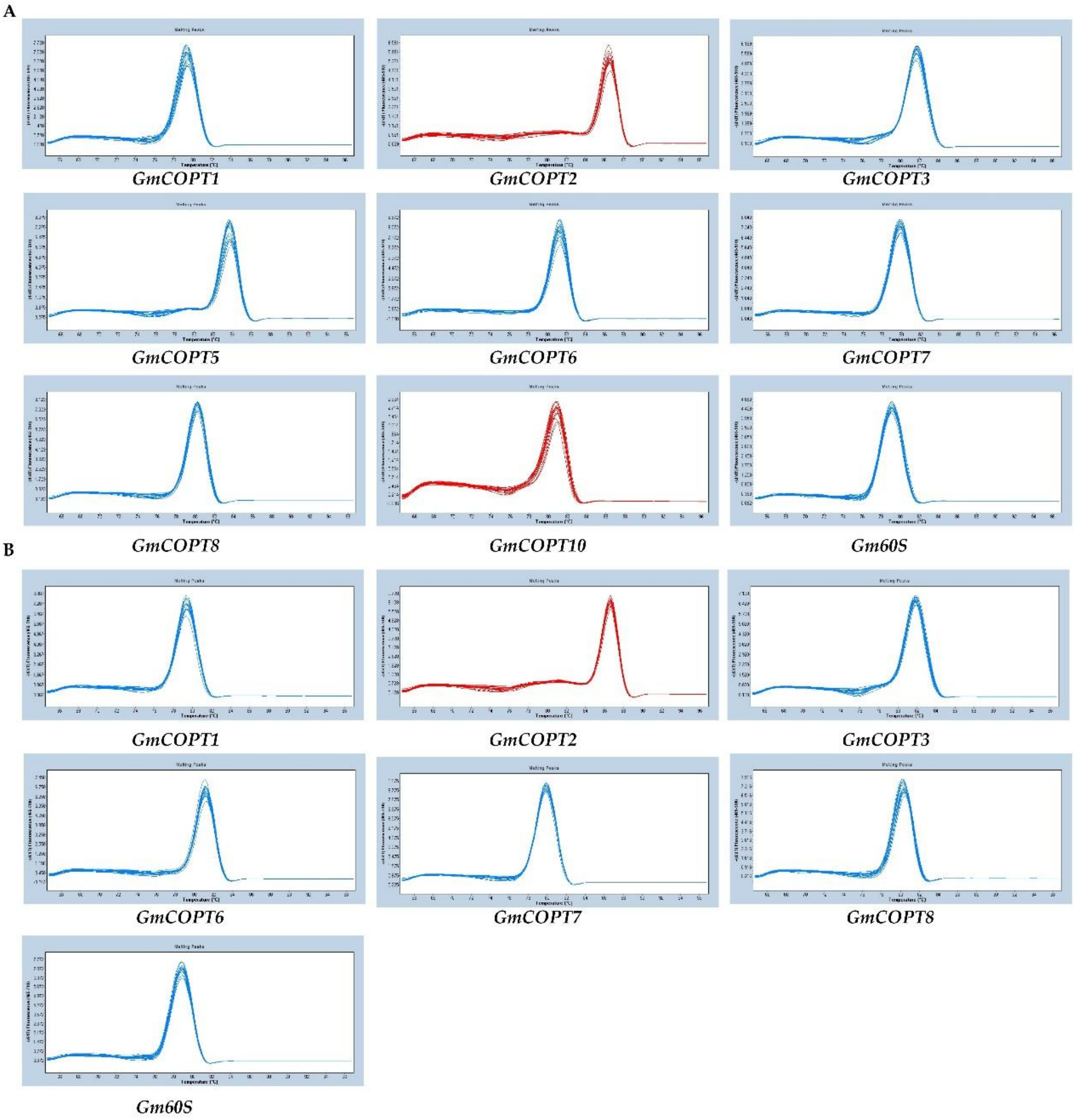

Figure S1. Melting curve analysis of the primer pairs used for RT-qPCR. (A) Melting curves of soybean genes from root samples. **(B)** Melting curves of soybean genes from leaf samples. A single sharp peak was observed for each amplicon, confirming specific amplification with no detectable non-specific products or primer dimers.
